# Synthesis and Surface-Enhanced Raman Scattering of Ultrathin SnSe_2_ Nanoflakes by Chemical Vapor Deposition

**DOI:** 10.3390/nano8070515

**Published:** 2018-07-10

**Authors:** Yongheng Zhang, Ying Shi, Meimei Wu, Kun Zhang, Baoyuan Man, Mei Liu

**Affiliations:** 1 School of Physics and Electronics, Shandong Normal University, Jinan 250014, China; zhangyongheng@stu.sdnu.edu.cn (Y.Z.); 2015020638@stu.sdnu.edu.cn (Y.S.); 2017020485@stu.sdnu.edu.cn (M.W.); 2016020591@stu.sdnu.edu.cn (K.Z.); byman@sdnu.edu.cn (B.M.); 2 Institute of Materials and Clean Energy, Shandong Normal University, Jinan 250014, China

**Keywords:** SnSe_2_, CVD, ultrathin, SERS

## Abstract

As a new atomically layered, two-dimensional material, tin (IV) diselenide (SnSe_2_) has attracted extensive attention due to its compelling application in electronics and optoelectronics. However, the great challenge of impurities and the preparation of high-quality ultrathin SnSe_2_ nanoflakes has hindered far-reaching research and SnSe_2_ practical applications so far. Therefore, a facile chemical vapor deposition (CVD) method is employed to synthesize large-scale ultrathin SnSe_2_ flakes on mica substrates using SnSe and Se powder as precursors. The structural characteristics and crystalline quality of the product were investigated. Moreover, Raman characterizations indicate that the intensity of A_1g_ peak and E_g_ peak, and the Raman shift of E_g_ are associated with the thickness of the SnSe_2_ nanoflakes. The ultrathin SnSe_2_ nanoflakes show a strong surface-enhanced Raman spectroscopy (SERS) activity for Rhodamine 6G (R6G) molecules. Theoretical explanations for the enhancement principle based on the chemical enhancement mechanism and charge transfer diagram between R6G and SnSe_2_ are provided. The results demonstrate that the ultrathin SnSe_2_ flakes are high-quality single crystal and can be exploited for microanalysis detection and optoelectronic application.

## 1. Introduction

Two-dimensional (2D) layered materials have received intensive attention owing to their superior properties in the field of photonics, electronics, and optoelectronics. The unique ultrathin 2D structure causes tunable band gaps and large specific surface areas [1,2,3]. Since graphene began to be explored [4], many 2D materials, such as transition metal sulfides (MoS_2_ [5,6], MoSe_2_ [7,8], and WSe_2_ [9,10]), III-VIA group semiconductors (GaS [11,12], InSe [13,14]), and IV-VIA group semiconductors (SnS_2_ [15,16]), have shown excellent performances in photodetectors [7], phototransistors [6], field effect transistors (FETs) [5], supercapacitor electrodes [17], and energy storage [18]. In recent decades, growing interest has focused on the exploration of 2D layered materials for potential usage in surface-enhanced Raman scattering [19]. Surface-enhanced Raman spectroscopy (SERS) is an important and powerful tool for the characterization of material structure. As a highly promising analytical tool, SERS plays an important role in ultrasensitive biomedical and chemical detection, which is very helpful for the potential application of chemical and biological sensing. The large specific surface area and specific structure have a tremendous influence on properties of 2D material. Accordingly, it is a pressing matter to synthesize large-scale ultrathin 2D layered materials and explore its Raman enhancement effect for further research.

Tin (IV) selenide (SnSe_2_), as a momentous material of the IV-VIA group, crystallizes with a hexagonal crystal structure in space group P-3m_1_ of the CdI_2_-type, in which the Sn–Se–Sn sandwich layered structure is formed by strong covalent forces in plane and weak van der Waals interaction dominates out of plane [20,21]. SnSe_2_ is a potential candidate for anodes for lithium-ion batteries [22], solar cells [23], supercapacitors [24], optoelectronic devices [25], and phase change memory [26] because of its appropriate indirect band gap (theoretical value of 0.71 eV in bulk material and 0.969 eV monolayer material). Moreover, both Sn and Se elements are earth-abundant and environmentally friendly, which further makes 2D SnSe_2_ a potential candidate for optoelectronics.

Exfoliation in bulk is an easy and convenient method to obtain single- or few-layered thin film for the most 2D layered materials, such as MoS_2_, graphene, and SnSe_2_ [20,27]. However, the exfoliated 2D material has limited applications due to poor control in size and low yield. Some other synthetic methods, such as hydrothermal synthesis [28,29], molecular beam epitaxy [30], and chemical vapor deposition (CVD) [25,31,32] have also been used for the synthesis of layered material. Among them, the CVD method has been proposed as an important and successful method to synthesize various single-crystalline ultrathin layered 2D materials, such as MoSe_2_, WSe_2_, and their heterostructures, due to the advantages of high yield and high crystal quality [33]. Motivated by this, He et al. reported the shape evolution of SnSe_2_ nanoflakes on SiO_2_/Si substrate via CVD employing Se and SnSe powder as precursors with the thinnest approximately 10 nm [25]. Then, Zhai et al. synthesized SnSe_2_ nanoflakes via CVD employing Se and SnI_2_ powder as precursors [21]. However, there are some inherent problems with this method, such as the use of tin sources which introduce new impurities. Therefore, more improvements are required to acquire large-scale, high-quality SnSe_2_ for practical applications.

In this work, we have successfully synthesized large-scale ultrathin SnSe_2_ nanoflakes of high quality on mica substrates employing Se and SnSe powder as precursors. SnSe with an appropriate melting point as a tin source does not introduce new impurities and guarantees the synthesis of ultrathin SnSe_2_ nanoflakes. Mica was selected as a substrate to guarantee the growth of SnSe_2_ along the horizontal orientation. Argon was used as the transfer gas to ensure the safety of the experiment. The lateral dimension of the nanoflakes is about 10 μm, and the layer number mainly varies from a monolayer to dozens of layers. In particular, various shapes of SnSe_2_, such as hexagonal, truncated triangular, and triangular were synthesized. Optical microscopy (OM), scanning electron microscopy (SEM), energy dispersive X-ray spectroscopy (EDX), transmission electron microscopy (TEM), X-ray photoelectron spectroscopy (XPS), X-ray diffraction (XRD), and atomic force microscopy (AFM) were employed to characterize the crystal quality, composition and crystal structures of the SnSe_2_ nanoflakes. Then, we further study the Raman peak change of sample with the decrease of thickness, and the Raman enhancement of SnSe_2_ on Rhodamine 6G (R6G) molecules. The ultrathin SnSe_2_ shows an excellent SERS activity for R6G, and the enhancement effect is explained by the principle of charge transfer.

## 2. Experimental Section

### 2.1. Synthesis of the SnSe_2_ Nanoflakes

The synthesis of the SnSe_2_ nanoflakes was carried out in a horizontal one-zone tube furnace (Lindberg/Blue M, TF55035KC-1, Thermo scientific, Asheville, NC, USA) with a silica tube (in a diameter of 1 inch and length of 20 inches) by CVD method. During the typical growth process, a high-purity Se powder (99.9% Alfa) (in one quartz boat) of 20 mg was placed at about 13.5 cm away from the center of the furnace and 10 mg of SnSe powder (99.9% Alfa) (in another quartz boat) was placed at the center of the furnace. Prior to the experiment, the two quartz boats were ultrasonically cleaned with acetone, ethanol and deionized water. Freshly cleaved mica substrate (10 mm × 20 mm Changchun Taiyuan Fluorphlogopite Co. Ltd., Changchun, China) was placed downstream of the tube furnace, and the distance between the mica and the center of the tube furnace was 13 cm. The temperature of the central zone increased from room temperature to 600 °C in 12 min to reduce chemical reactions during heating. The growth process lasted 5 min at a total pressure of 120 Torr and an argon atmosphere of 30 standard cubic centimeters per minute (sccm). Once the reaction was achieved, the lid of the furnace was open immediately for the purpose of cooling. Triangular truncated, triangular and hexagonal SnSe_2_ nanoflakes were deposited on the mica substrate.

### 2.2. Characterization of SnSe_2_ Nanoflakes

The morphology and size of as-synthesized SnSe_2_ flakes were characterized by an OM (Axio Observer. A1m, Zeiss, Oberkochen, Germany) and SEM (Sigma 500, Zeiss, Oberkochen, Germany). An AFM (Dimension Icon, Bruker, Karlsruhe, Germany) was used to measure the morphology and thickness of the SnSe_2_ flakes. The crystalline structure and composition of the SnSe_2_ were investigated by adopting XRD, XPS, TEM, and EDX. TEM, High-resolution TEM imaging, and selected area electron diffraction (SAED) (JEM-2100, Japan Electron Optics Laboratory Co. Ltd., Tokyo, Japan) measurements were performed on the TEM grid by transferring with poly(methyl methacrylate) (PMMA) method. An EDX (XFlash 6130, Bruker, Berlin, Germany) was used to characterize element ratio and uniformity. The information of binding energies in SnSe_2_ was characterized by employing an XPS (Escalab 250, Thermo Fisher scientific, Waltham, MA, USA) to verify the presence of Sn and Se. Crystal faces were identified and scanned by using an XRD (EPMA-1600, ShimaDu, Tokyo, Japan) with the angular range of 2θ from 10° to 80°. Raman spectra were recorded in a confocal Raman system (HR Evolution, Horiba, Paris, France) with a 532 nm laser, the laser spot was ~1 μm and the powder on the sample surface was kept below 0.5 mw to avoid any heating effects. Raman mapping was collected by a confocal Raman system with a laser wavelength of 532 nm and scanning step intervals of 1 μm.

## 3. Results and Discussions

Typically, ultrathin SnSe_2_ nanoflakes were synthesized on mica substrates at 600 °C with Ar (30 sccm) flows under the pressure of 120 Torr via CVD method (for more details see the Experimental Section). In the process of growth, SnSe and Se powder, used as precursors, were put in the two different quartz boats, respectively. In comparison with other sources, such as SnO_2_ SnI_2_ and Sn, SnSe was used as Sn source because its proper melting point (861 °C) guaranteed slow evaporation during the growth process. Moreover, the two precursors do not contain other elements of SnSe_2_, resulting in new impurities [32]. In addition, fluorophlogopite mica was utilized as the substrate for growing 2D materials due to its chemical inertness and atomically smooth surface [34]. Furthermore, SnSe_2_ grown on mica substrate was expected to obtain in-plane flakes due to mica having the van der Waals substrate (graphene, SnSe_2_, etc.), which could freely release the strain energy [16].

**Figure 1:**
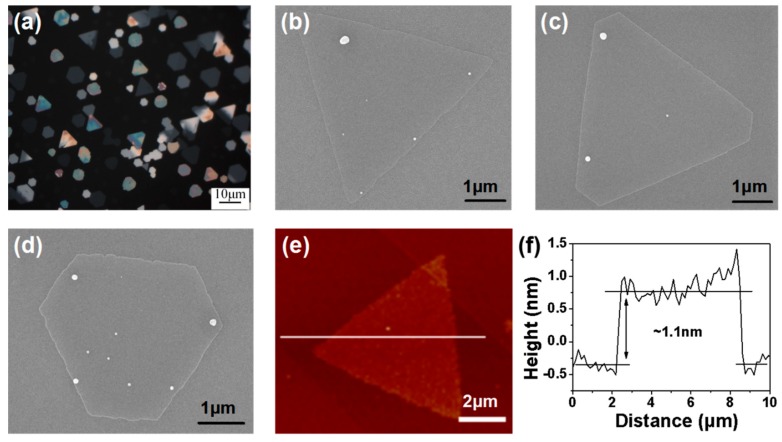
Morphology of as-synthesized atomic-layer SnSe_2_ nanoflakes. (**a**) Large-scale optical microscopy (OM) image of as-grown SnSe_2_ nanoflakes on mica to show high yield of SnSe_2_; Typical scanning electron microscopy (SEM) images of SnSe_2_ single crystal with (**b**) triangle; (**c**) truncated triangle, and (**d**) hexagon, respectively; (**e**) Atomic force microscopy (AFM) image of a typical SnSe_2_ nanoflake; (**f**) The height profile in (**e**) indicates that the SnSe_2_ nanoflakes are monolayer.

OM, SEM, and AFM were used to measure the morphology of the SnSe_2_ nanoflakes grown by CVD method. Figure 1a shows a typical optical image of large-scale and ultrathin SnSe_2_ flakes with lateral lengths of about 10 µm. Moreover, large-scale optical images show a high-yield of SnSe_2_ nanoflakes (Figure S1, Supporting Information). The images show that SnSe_2_ nanoflakes with different shapes including hexagonal, truncated triangular, and triangular, are distributed on the mica substrates. The shape evolution of SnSe_2_ nanoflakes may be slightly different from the ratio of elements [25]. Nanoflakes with different thicknesses observed by OM showed slightly different colors. To sum up, thinner SnSe_2_ nanoflakes have darker color (dark), and thicker nanoflakes have brighter color (white) [35]. The color contrast indicates that the thickness of the SnSe_2_ nanoflakes varies slightly between different nanoflakes. The magnified SEM images (Figure 1b–d) show that the SnSe_2_ samples have regular shapes and smooth surfaces except for some points. The thicknesses of the SnSe_2_ nanoflakes were further characterized by AFM. The AFM image (Figure 1e) and the corresponding step height profile curve (Figure 1f) of a SnSe_2_ nanoflake reveals a relatively flat surface with a thickness of about 1.1 nm, such thickness corresponds to monolayers [20]. More height measurements demonstrate that the thickness of the SnSe_2_ nanoflakes is mainly between 1.1 nm to 10 nm (Figure S2, Supporting Information).

**Figure 2:**
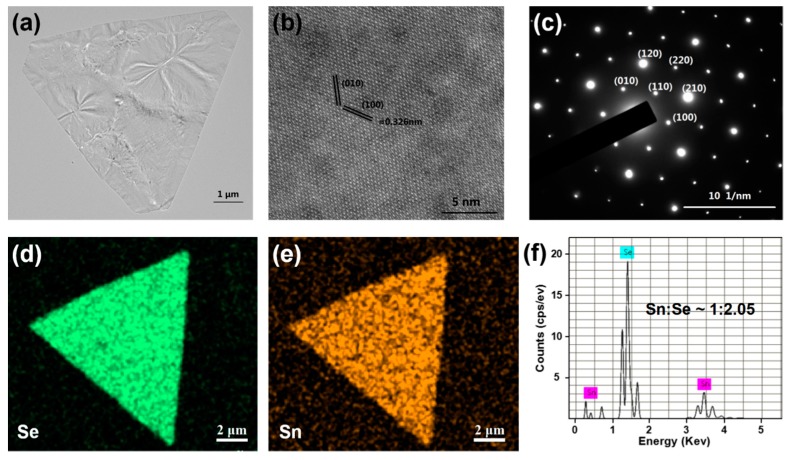
Transmission electron microscopy (TEM) and energy dispersive X-ray spectroscopy (EDX) characterization of SnSe_2_ nanoflakes. (**a**) Low-magnification TEM image of the typical SnSe_2_ nanoflake; (**b**) The corresponding high-resolution TEM image of the SnSe_2_ nanoflake in (a); (**c**) The selected area electron diffraction pattern of the SnSe_2_ single crystal; (**d**,**e**) The Se and Sn elemental mapping of the SnSe_2_ nanoflakes on mica, respectively; (**f**) EDS spectrum of the as-grown SnSe_2_ nanoflake on mica.

The crystal quality of the SnSe_2_ nanoflake was characterized by TEM and the selected area electron diffraction (SAED) pattern. The SnSe_2_ samples were transferred to a TEM grid with the help of poly(methyl methacrylate) (PMMA) [36]. Figure 2a shows a low-magnification TEM image of a representative SnSe_2_ nanoflake. The invariant of the transferred sample indicates the high-quality SnSe_2_ produced by CVD growth. Contrast SEM images, the samples observed with TEM under the same magnification have folds, indicating that folds are caused during the transfer and drying process [37]. The corresponding high-resolution TEM (HRTEM) image (Figure 2b) shows obvious lattice fringe, and the lattice spacing of 0.326 nm is in accordance with the (100) interplanar distance of the SnSe_2_, and the perfect lattice fringe suggests the high-quality of our samples [29]. Moreover, the SAED pattern is presented in Figure 2c, showing the hexagonal crystalline of SnSe_2_, clearly reveals a six-fold symmetry similar to other 2D layered materials, such as MoS_2_ [38,39]. These results indicate that our SnSe_2_ is a hexagonal high-quality single crystal. To further identify the composition and uniformity of the sample, we employed the EDX mapping, shown in Figure 2d,e, to explore the uniformity of spatial distribution of Se and Sn elements across the whole SnSe_2_ nanoflake. In addition, the corresponding EDX spectrum of the SnSe_2_ nanoflake is shown in Figure 2f. The atomic ratio of Se and Sn is about 2:1, coinciding well with the element ratio of SnSe_2_. These characterization data indicate that the product is highly crystalline SnSe_2_ crystal without impurities.

**Figure 3:**
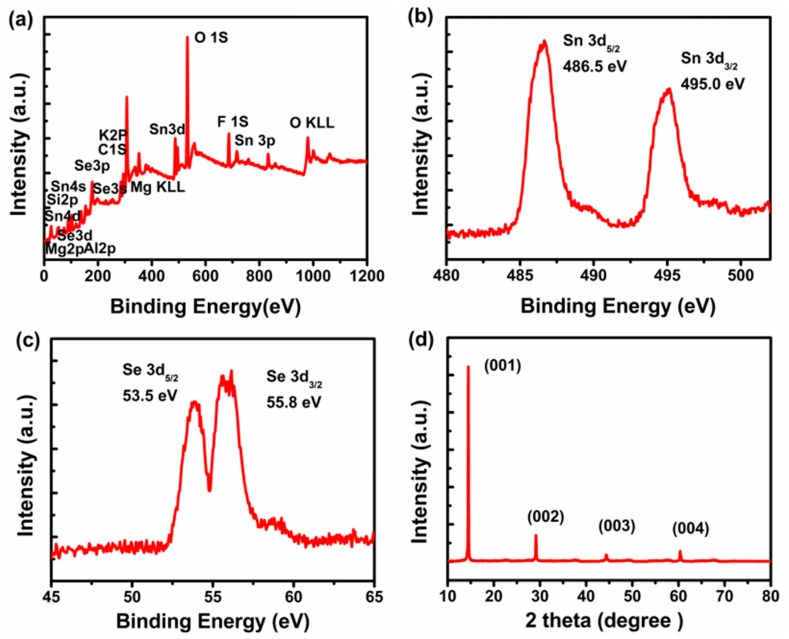
X-ray photoelectron spectroscopy (XPS) and X-ray diffraction (XRD) characterization of the SnSe_2_ crystal on mica. (**a**) The whole range of an XPS spectrum of the SnSe_2_; (**b**,**c**) XPS peak of Sn and Se elements in the SnSe_2_ crystal, respectively; (**d**) XRD pattern of the SnSe_2_ crystal.

The chemical compositions and bonding types of the SnSe_2_ nanoflakes grown on mica by CVD method were examined with XPS. It provides the information on stoichiometry and bonding of samples. Eight elements are present in the spectra shown in Figure 3a. Sn and Se elements come from the SnSe_2_ nanoflakes, and others come from the mica (KMg_3_(AlSi_3_O_10_)F_2_) substrate. The 3d spectra of the SnSe_2_ samples (Figure 3b,c) have Sn3d_3/2_, Sn3d_5/2_, Se3d_3/2_, and Se3d_5/2_ peaks which can be observed at 495.0 eV, 486.5 eV, 55.8 eV and 53.5 eV, respectively [40]. Sn3d_3/2_ and Sn3d_5/2_ characteristic peaks are situated at 495.0 and 486.5 eV, respectively, certifying that tin is in its Sn (IV) state [41,42]. The binding energies of 55.8 and 53.5 eV are attributed to the doublet Se3d_3/2_ and Se3d_5/2_ peaks corresponding to the −2 state of Selenium, which coincides with the values of the previous work [43]. The characteristic peaks of tin indicate that the valence state of Sn changes from Sn^2+^ to Sn^4+^ [42]. The peaks of Se at about 55 eV were split into Se3d_5/2_ (53.5 eV) and Se3d_3/2_ (55.8 eV) [43]. The XPS further suggests that the products are SnSe_2_ without other impurities, such as SnSe.

XRD patterns were characterized to elucidate the overall crystallinity on a mica substrate. The XRD pattern obtained from SnSe_2_ nanoflakes is shown in Figure 3d. The XRD pattern of as-synthesized crystal is very consistent with the characteristic peaks of standard SnSe_2_. The prominent peaks at 2θ values of 14°, 29°, 44.2°, and 60°, correspond to the (001), (002), (003), and (004) planes of SnSe_2_, respectively [29,31,41]. This shows that the SnSe_2_ has a hexagonal lattice structure. The most obvious peak is located at 2θ = 14°, corresponding to the (001) crystal plane, revealing that the SnSe_2_ crystal grain preferentially grows along the *c*-axis. In addition, the obtained nanoflakes have a hexagonal lattice with the lattice constant a = b = 0.38 nm and c = 0.614 nm that agrees with the JCPDSN 89-2939 for the referred SnSe_2_ crystal. In brief, the XRD patterns coincide with the literature data of the characteristic peaks of SnSe_2_ crystal and prove that the as-grown products are made up of SnSe_2_.

**Figure 4:**
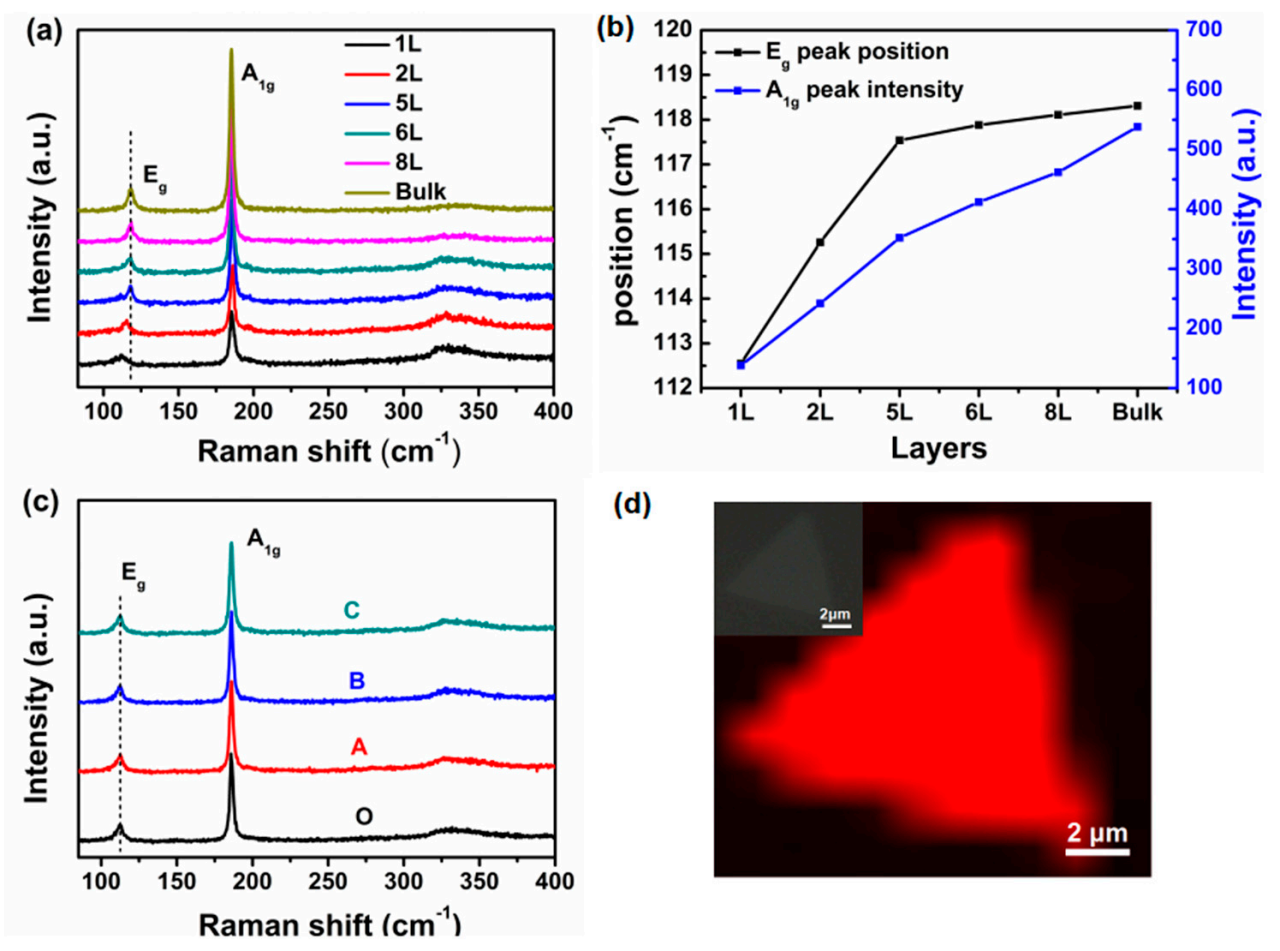
Raman characterization of the SnSe_2_ nanoflakes. (**a**) Thickness-dependent Raman spectra of the as-synthesized SnSe_2_ nanoflakes on mica substrate; (**b**) The position of the E_g_ peak and the intensity of the A_1g_ peak versus number of SnSe_2_ layers; (**c**) Raman spectra from four different positions of a triangular sample (corresponding Raman optical image in Figure S3); (**d**) Raman mapping of A_1g_ mode of a typical triangular SnSe_2_ nanoflake. Inset: corresponding Raman optical image.

Raman spectra were employed to investigate the crystal structure of the as-synthesized SnSe_2_ nanoflakes thoroughly. As far as we know, the peak of SnSe_2_ in about 185 cm^−1^ is assigned to out-plane vibrational A_1g_ mode; in the light of the E_g_ peak position, tin selenide can be divided into two different polytypes: 2H and 1T. For the 2H-SnSe_2_ crystal, the E_g_ mode is located at 108 cm^−1^ approximately. For the 1T-SnSe_2_ crystal, the E_g_ mode gives rise to a band at 118 cm^−1^ [44]. As we observed in Figure 4a, the Raman spectra of the SnSe_2_ nanoflakes show a distinct identification of 1T-SnSe_2_ Raman spectra. With the decrease in thickness, the E_g_ peak becomes inconspicuous due to the reduction in the number of scattering centers in the ultrathin SnSe_2_ nanoflakes, which is in agreement with the previous literature [45]. The change of E_g_ peak position with the thickness of SnSe_2_ is shown in Figure 4b, in which an obvious redshift is observed. The wavenumber of the E_g_ mode decreases slightly from 118.3 cm^−1^ to 112.6 cm^−1^ with the reduction in the layer number, which can be attributable to surface effects and inter- and intralayer couplings [46]. The intensity of out-plane vibration mode (A_1g_) decreases remarkably as the thickness decreases, which is likely induced by the mechanical properties of the SnSe_2_ nanoflakes. For 2D layered material, the layers are coupled together by van der Waals forces. With the increase of the layer number, the van der Waals forces increase quickly, which can be reflected by the variation of Young’s modulus of crystalline 2D flakes [37]. The increase of out-plane vibration will influence the corresponding Raman intensity. This phenomenon, perhaps, provides an easy way to judge the number of the SnSe_2_ layers on mica substrates. In addition, the Raman spectra (Figure 4c) of four different points from a SnSe_2_ nanoflake show the identical feature of 1T-phase, revealing the homogeneity of the as-grown SnSe_2_ (Figure S3, Supporting Information). Moreover, Raman intensity mapping of the A_1g_ mode of the typical SnSe_2_ nanoflakes at 185 cm^−1^ reveals a uniform intensity over the whole flake (Figure 4d). Moreover, the Raman peaks of SnSe have not been found [25], indicating that high purity SnSe_2_ is successfully synthesized via the improved CVD method.

In a word, the synthesis of high-quality SnSe_2_ was obtained by using mica substrate and SnSe source. SnSe with an appropriate melting point is beneficial to the synthesis of ultrathin SnSe_2_ and does not introduce new impurities. As a van der Waals substrate, mica is propitious to the horizontal growth of the crystal. Simultaneously, the tube furnace was heated to 600 °C in 12 min to reduce the reaction of the source during the heating process. The pressure was maintained at 120 Torr to ensure the formation of ultrathin samples. The pressure was too high to grow into a thicker sample, as shown in Figure S4. Argon was used as transmission gas, which is convenient to operate and ensures the safety of the experiment. Then, the characterizations of the morphology and structure of the samples indicate that the as-grown SnSe_2_ is a pure phase, high-quality, single crystal.

**Figure 5:**
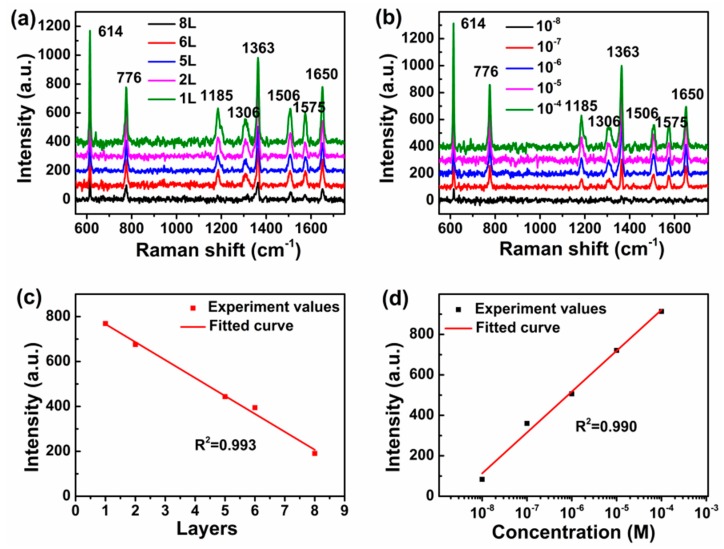
(**a**) Surface-enhanced Raman spectroscopy (SERS) spectra of 1 × 10^−4^ M Rhodamine 6G (R6G) molecule absorbed on different thickness SnSe_2_ nanoflakes; (**b**) SERS spectra of R6G with concentration from 10^−4^ to 10^−8^ M on monolayer SnSe_2_ nanoflakes; (**c**) The SERS intensity at 614 cm^−1^ of R6G as a function of the thickness of SnSe_2_ nanoflakes; (**d**) The intensity of SERS peak at 614 cm^−1^ of R6G changes as a function of the molecular concentration on the SnSe_2_/mica substrate. Laser wavelength: 532 nm; power: 0.5 mw; lens: 100 × objective; acquisition time: 8 s.

To prove the potential application in microanalytics and optoelectronics, SERS effect of R6G molecules on the different thicknesses of SnSe_2_ was analyzed. Herein, the main vibrations of R6G molecule are confirmed according to the previous article [47]. As shown in Figure 5a, the two strong vibration modes of 614 and 776 cm^−1^ are due to vibronic coupling between R6G molecules and SnSe_2_; the four high-frequency vibration modes are caused by the stretching of aromatic C–C; the remaining two modes are attributable to the combination of the four stretching modes. The Raman spectra of R6G molecules on SnSe_2_ with different layers are shown in Figure 5a. The Raman spectral peaks of R6G on monolayer SnSe_2_ were significantly higher than the other layers of SnSe_2_. All identifiable vibration modes of R6G decrease with the increase in the number of SnSe_2_ layers. Two vibrational peaks at 1185 and 1306 cm^−1^ disappear until R6G molecules are deposited on eight layers of SnSe_2_, which is probably because the two vibration modes are relatively weak. The relationship between R6G signal intensity and thickness of SnSe_2_ indicates that the thickness of the SnSe_2_ seriously affects SERS activities. To explore the enhanced ability of different thicknesses of SnSe_2_ substrates, the linear fitted curve of the relationship between the number of SnSe_2_ layers and the intensity of the peak at 614 cm^−1^ is presented in Figure 5c. A linear response with a high coefficient of determination (R^2^: 0.993) is achieved. In addition, SERS measurements were carried out under different concentrations of R6G molecules (Figure 5b). The Raman intensity value of the vibrational peak at 1363 cm^−1^ of R6G with 10^−6^ M concentration taken for SnSe_2_ substrate is larger than those obtained for R6G on other substrates, such as WSe_2_, Graphene, MoS_2_ (about 2.5-, 2.5-, and 10-fold higher than WSe_2_, Graphene, and MoS_2_, respectively) [48]. These results may be due to the lower quantum yield collected from other materials compared with that obtained from SnSe_2_. When the concentration of R6G is as low as 10^−7^ M, the Raman peaks (614, 776, 1185, 1306, 1363, 1506, 1575, and 1650 cm^−1^) are still conspicuous. Specifically, when the concentration of R6G is lowered to 10^−8^ M, the vibrational peak at 614 cm^−1^ still exists because of the existence of strong vibronic coupling between SnSe_2_ and R6G molecules. Herein, we investigated the relationship between the intensity of 614 cm^−1^ peak and the R6G concentration. The consummate linear response of the SnSe_2_/mica substrate with the high determination coefficient (R^2^: 0.990) in log scale is shown in Figure 5d. In general, these results demonstrate that the as-synthesized SnSe_2_ has a strong effect on the R6G molecule and expand the application of SnSe_2_ to microanalysis and microdetection.

For the Raman enhancement mechanism, the electromagnetic mechanism (EM) and chemical mechanism (CM) are two widely accepted mechanisms [49]. EM is mainly due to the enhancement of the local electromagnetic field caused by light-excited surface plasmons resonance. The EM mechanism requires the surface of the substrates to be rough and, therefore, generate surface plasmons resonance. CM is caused by photoinduced charge transfer between the target molecule and substrate. During the charge transfer process, the polarizability of molecules increases, which effectively enhances the recombination of electrons and holes as well as the Raman scattering intensity. Furthermore, the CM mechanism requires a smooth surface and the Fermi level of the substrate is located between the highest occupied molecular orbit (HOMO) and the lowest unoccupied molecular orbit (LUMO). Charge transfer is able to happen from the substrate to the molecule or vice versa. After careful consideration of the specific surface area and relatively smooth surface of 2D SnSe_2_, the CM between the SnSe_2_ and the R6G molecule is evaluated.

**Scheme 1:**
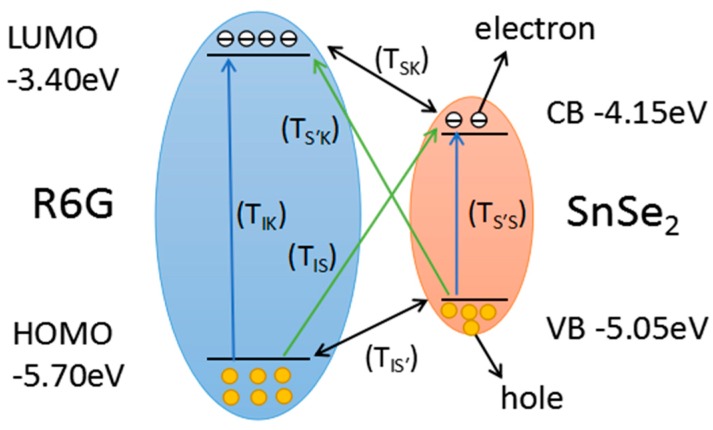
Schematic energy diagram illustrating charge transfer process between R6G molecule and SnSe_2_ under the excitation of 532 nm.

Scheme 1 describes the charge-transfer process at the interface between R6G target molecule and SnSe_2_ substrate. As a traditional SERS probe, for R6G molecules, the HOMO is at −5.70 eV, and LUMO is at −3.40 eV. The valence band (VB) (−5.05 eV) and conduction band (CB) (−4.95 eV) of SnSe_2_ are located between the HOMO and LUMO. The vibronic coupling between SnSe_2_ (CB state |S> and VB state |S′>) and R6G molecule (excited state |K> and ground state |I>) leads to the charge transfer process. With consideration of the excitation energy and R6G molecule, when we use a laser with a laser wavelength of 532 nm, the following transfer processes can occur according to the energy level relationship: (1) (T_S′S_), (2) (T_IK_), (3) (T_S′K_), (4) (T_IS_), (5) (T_IS′_) and (6) (T_SK_) which are in shown in Scheme 1. These enormously enhance the polarizability of the R6G molecule and result in an increase in recombination of electrons and holes, and then enhance the peak intensity of R6G molecules. As a result of the CM mechanism, charge transfer is most likely to occur between the R6G molecule and a layer of SnSe_2_ that is in contact with molecules [50]. With the increase in the number of SnSe_2_ layers, the enhancement effect becomes weaker which can be associated with the band gap. The theoretical energy band gap values of SnSe_2_ [20] show that the band gap becomes larger with the reduction of thickness, which may lead to a decrease in the chemical potential difference between R6G molecule and SnSe_2_ and then the increase of the charge transfer. In brief, these results prove that the Raman enhancement mechanism in the SnSe_2_ is basically related to the charge transfer theory given the energy band structure.

## 4. Conclusions

In summary, we report the growth of large-scale, high-quality, ultrathin SnSe_2_ flakes on mica substrates via an improved chemical vapor deposition. The typical final product shows triangular, truncated triangular and hexagonal shapes with lateral size ranging from a few microns to a dozen microns and its smallest thickness of ~1.1 nm (monolayer). A variety of detailed characterization demonstrated that the synthesized SnSe_2_ flakes were high-quality, single crystals with the growth orientation along *c*-axis. Moreover, Raman spectra of the SnSe_2_ flakes of different thickness reveal that an obvious redshift for E_g_ mode with the reduction of the layer number but with no change for A_1g_ mode. Furthermore, we investigated the SERS of R6G molecules on SnSe_2_ flakes, and this phenomenon was explained based on the principle of charge transfer. We have demonstrated that the as-synthesized SnSe_2_ is a promising 2D material in microanalysis filed.

## Supplementary Materials

The following are available online at http://www.mdpi.com/2079-4991/8/7/515/s1, Figure S1: Large-scale optical images of the as-synthesized SnSe2 nanoflakes, Figure S2: AFM images of SnSe2 nanoflakes with different thickness. The insets show the corresponding height profiles, Figure S3: The Raman optical image of triangular nanoflakes, The Raman spectra corresponding to four positions are found in Figure 4c, Figure S4: SEM image of SnSe2 nanoflakes in higher pressure.

## References

[1] Wang Q.H., Kalantar-Zadeh K., Kis A., Coleman J.N., Strano M.S. (2012). Electronics and optoelectronics of two-dimensional transition metal dichalcogenides. Nat. Nanotechnol..

[2] Mak K.F., Lee C., Hone J., Shan J., Heinz T.F. (2010). Atomically thin MoS_2_: A new direct-gap semiconductor. Phys. Rev. Lett..

[3] Ji Q., Zhang Y., Zhang Y., Liu Z. (2015). Chemical vapour deposition of group-VIB metal dichalcogenide monolayers: Engineered substrates from amorphous to single crystalline. Chem. Soc. Rev..

[4] Novoselov K.S., Geim A.K., Morozov S.V., Jiang D., Zhang Y., Dubonos S.V., Grigorieva I.V., Firsov A.A. (2004). Electric field effect in atomically thin carbon films. Science.

[5] Van der Zande A.M., Huang P.Y., Chenet D.A., Berkelbach T.C., You Y., Lee G.H., Heinz T.F., Reichman D.R., Muller D.A., Hone J.C. (2013). Grains and grain boundaries in highly crystalline monolayer molybdenum disulphide. Nat. Mater..

[6] Choi W., Cho M.Y., Konar A., Lee J.H., Cha G.B., Hong S.C., Kim S., Kim J., Jena D., Joo J. (2012). High-detectivity multilayer MoS_2_ phototransistors with spectral response from ultraviolet to infrared. Adv. Mater..

[7] Xia J., Huang X., Liu L.Z., Wang M., Wang L., Huang B., Zhu D.D., Li J.J., Gu C.Z., Meng X.M. (2014). CVD synthesis of large-area, highly crystalline MoSe_2_ atomic layers on diverse substrates and application to photodetectors. Nanoscale.

[8] Baek J., Yin D., Liu N., Omkaram I., Jung C., Im H., Hong S., Kim S.M., Hong Y.K., Hur J. (2016). Highly sensitive chemical gas detecting transistor based on highly crystalline CVD-grown MoSe_2_ films. Nano Res..

[9] Gao Y., Hong Y.-L., Yin L.-C., Wu Z., Yang Z., Chen M.-L., Liu Z., Ma T., Sun D.-M., Ni Z. (2017). Ultrafast growth of high-quality monolayer WSe_2_ on Au. Adv. Mater..

[10] Huang J.-K., Pu J., Hsu C.-L., Chiu M.-H., Juang Z.-Y., Chang Y.-H., Chang W.-H., Iwasa Y., Takenobu T., Li L.-J. (2013). Large-area synthesis of highly crystalline WSe_2_ monolayers and device applications. ACS Nano.

[11] Late D.J., Liu B., Luo J., Yan A., Matte H.S., Grayson M., Rao C.N., Dravid V.P. (2012). GaS and GaSe ultrathin layer transistors. Adv. Mater..

[12] Hu P., Wang L., Yoon M., Zhang J., Feng W., Wang X., Wen Z., Idrobo J.C., Miyamoto Y., Geohegan D.B. (2013). Highly responsive ultrathin GaS nanosheet photodetectors on rigid and flexible substrates. Nano Lett..

[13] Feng W., Zheng W., Cao W., Hu P. (2014). Back gated multilayer InSe transistors with enhanced carrier mobilities via the suppression of carrier scattering from a dielectric interface. Adv. Mater..

[14] Feng W., Wu J.-B., Li X., Zheng W., Zhou X., Xiao K., Cao W., Yang B., Idrobo J.-C., Basile L. (2015). Ultrahigh photo-responsivity and detectivity in multilayer InSe nanosheets phototransistors with broadband response. J. Mater. Chem. C.

[15] Su G., Hadjiev V.G., Loya P.E., Zhang J., Lei S., Maharjan S., Dong P., P M.A., Lou J., Peng H. (2015). Chemical vapor deposition of thin crystals of layered semiconductor SnS_2_ for fast photodetection application. Nano Lett..

[16] Yang D., Li B., Hu C., Deng H., Dong D., Yang X., Qiao K., Yuan S., Song H. (2016). Controllable growth orientation of SnS_2_ flakes for low-noise, high-photoswitching ratio, and ultrafast phototransistors. Adv. Opt. Mater..

[17] Acerce M., Voiry D., Chhowalla M. (2015). Metallic 1T phase MoS_2_ nanosheets as supercapacitor electrode materials. Nat. Nanotechnol..

[18] Huang X., Zeng Z., Zhang H. (2013). Metal dichalcogenide nanosheets: Preparation, properties and applications. Chem. Soc. Rev..

[19] Fiori G., Bonaccorso F., Iannaccone G., Palacios T., Neumaier D., Seabaugh A., Banerjee S.K., Colombo L. (2014). Electronics based on two-dimensional materials. Nat. Nanotechnol..

[20] Yu P., Yu X., Lu W., Lin H., Sun L., Du K., Liu F., Fu W., Zeng Q., Shen Z. (2016). Fast photoresponse from 1T tin diselenide atomic layers. Adv. Funct. Mater..

[21] Zhou X., Gan L., Tian W., Zhang Q., Jin S., Li H., Bando Y., Golberg D., Zhai T. (2015). Ultrathin SnSe_2_ flakes grown by chemical vapor deposition for high-performance photodetectors. Adv. Mater..

[22] Choi J., Jin J., Jung I.G., Kim J.M., Kim H.J., Son S.U. (2011). SnSe_2_ nanoplate-graphene composites as anode materials for lithium ion batteries. Chem. Commun..

[23] Yu X., Zhu J., Zhang Y., Weng J., Hu L., Dai S. (2012). SnSe_2_ quantum dot sensitized solar cells prepared employing molecular metal chalcogenide as precursors. Chem. Commun..

[24] Zhang C., Yin H., Han M., Dai Z., Pang H., Zheng Y., Lan Y.-Q., Bao J., Zhu J. (2014). Two-dimensional tin selenide nanostructures for flexible all-solid-state supercapacitors. ACS Nano.

[25] Huang Y., Xu K., Wang Z., Shifa T.A., Wang Q., Wang F., Jiang C., He J. (2015). Designing the shape evolution of SnSe_2_ nanosheets and their optoelectronic properties. Nanoscale.

[26] Wang R.Y., Caldwell M.A., Jeyasingh R.G.D., Aloni S., Shelby R.M., Wong H.S.P., Milliron D.J. (2011). Electronic and optical switching of solution-phase deposited SnSe_2_ phase change memory material. J. Appl. Phys..

[27] Zhou W., Yu Z., Song H., Fang R., Wu Z., Li L., Ni Z., Ren W., Wang L., Ruan S. (2017). Lattice dynamics in monolayer and few-layer SnSe_2_. Phys. Rev. B.

[28] Liu K., Liu H., Wang J., Feng L. (2009). Synthesis and characterization of SnSe_2_ hexagonal nanoflakes. Mater. Lett..

[29] Fang Z., Hao S., Long L., Fang H., Qiang T., Song Y. (2014). The enhanced photoelectrochemical response of SnSe_2_ nanosheets. CrystEngComm.

[30] Park Y.W., Jerng S.-K., Jeon J.H., Roy S.B., Akbar K., Kim J., Sim Y., Seong M.-J., Kim J.H., Lee Z. (2016). Molecular beam epitaxy of large-area SnSe_2_ with monolayer thickness fluctuation. 2D Mater..

[31] Wu J., Hu Z., Jin Z., Lei S., Guo H., Chatterjee K., Zhang J., Yang Y., Li B., Liu Y. (2016). Spiral growth of SnSe_2_ crystals by chemical vapor deposition. Adv. Mater. Interfaces.

[32] Huang L., Yu Y., Li C., Cao L. (2013). Substrate mediation in vapor deposition growth of layered chalcogenide nanoplates: A case study of SnSe_2_. J. Phys. Chem. C.

[33] Gong Y., Lei S., Ye G., Li B., He Y., Keyshar K., Zhang X., Wang Q., Lou J., Liu Z. (2015). Two-step growth of two-dimensional WSe_2_/MoSe_2_ heterostructures. Nano Lett..

[34] Peng H., Dang W., Cao J., Chen Y., Wu D., Zheng W., Li H., Shen Z.X., Liu Z. (2012). Topological insulator nanostructures for near-infrared transparent flexible electrodes. Nat. Chem..

[35] Hu Y., Chen T., Wang X., Ma L., Chen R., Zhu H., Yuan X., Yan C., Zhu G., Lv H. (2017). Controlled growth and photoconductive properties of hexagonal SnS_2_ nanoflakes with mesa-shaped atomic steps. Nano Res..

[36] Ma D., Shi J., Ji Q., Chen K., Yin J., Lin Y., Zhang Y., Liu M., Feng Q., Song X. (2015). A universal etching-free transfer of MoS_2_ films for applications in photodetectors. Nano Res..

[37] Sahabudeen H., Qi H., Glatz B.A., Tranca D., Dong R., Hou Y., Zhang T., Kuttner C., Lehnert T., Seifert G. (2016). Wafer-sized multifunctional polyimine-based two-dimensional conjugated polymers with high mechanical stiffness. Nat. Commun..

[38] Lee Y.H., Zhang X.Q., Zhang W., Chang M.T., Lin C.T., Chang K.D., Yu Y.C., Wang J.T., Chang C.S., Li L.J. (2012). Synthesis of large-area MoS_2_ atomic layers with chemical vapor deposition. Adv. Mater..

[39] Zhan Y., Liu Z., Najmaei S., Ajayan P.M., Lou J. (2012). Large-area vapor-phase growth and characterization of MoS_2_ atomic layers on a SiO_2_ substrate. Small.

[40] De Groot C.H., Gurnani C., Hector A.L., Huang R., Jura M., Levason W., Reid G. (2012). Highly selective chemical vapor deposition of tin diselenide thin films onto patterned substrates via single source diselenoether precursors. Chem. Mater..

[41] Saha S., Banik A., Biswas K. (2016). Few-layer nanosheets of n-type SnSe_2_. Chem.-Eur. J..

[42] Velicky M., Toth P.S., Rakowski A.M., Rooney A.P., Kozikov A., Woods C.R., Mishchenko A., Fumagalli L., Yin J., Zolyomi V. (2017). Exfoliation of natural van der waals heterostructures to a single unit cell thickness. Nat. Commun..

[43] Harbec J.Y., Powell B.M., Jandl S. (1983). Lattice dynamics of SnSe_2_. Phys. Rev. B.

[44] Smith A., Meek P., Liang W. (1977). Raman scattering studies of SnS_2_ and SnSe_2_. J. Phys. C Solid State Phys..

[45] Hu P., Wen Z., Wang L., Tan P., Xiao K. (2012). Synthesis of few-layer GaSe nanosheets for high performance photodetectors. ACS Nano.

[46] Lorchat E., Froehlicher G., Berciaud S. (2016). Splitting of interlayer shear modes and photon energy dependent anisotropic Raman response in n-layer ReSe_2_ and ReS_2_. ACS Nano.

[47] Michaels A.M., Jiang J., Brus L. (2000). Ag nanocrystal junctions as the site for surface-enhanced Raman scattering of single rhodamine 6G molecules. J. Phys. Chem. B.

[48] Lee Y., Kim H., Lee J., Yu S.H., Hwang E., Lee C., Ahn J.-H., Cho J.H. (2015). Enhanced Raman scattering of rhodamine 6G films on two-dimensional transition metal dichalcogenides correlated to photoinduced charge transfer. Chem. Mater..

[49] Campion A., Kambhampati P. (1998). Surface-enhanced Raman scattering. Chem. Soc. Rev..

[50] Ling X., Xie L., Fang Y., Xu H., Zhang H., Kong J., Dresselhaus M.S., Zhang J., Liu Z. (2010). Can graphene be used as a substrate for Raman enhancement?. Nano Lett..

