# Supplementary material for: Synthesis and Surface-Enhanced Raman Scattering of Ultrathin SnSe2 Nanoflakes by Chemical Vapor Deposition
Source: Nanomaterials (Basel). 2018 Jul 10;8(7):515. doi: 10.3390/nano8070515 (PMC6070886; doi:10.3390/nano8070515)
Supplement: Supplementary file 1 [file nanomaterials-08-00515-s001.pdf]

Supplementary Materials

Synthesis and Surface-Enhanced Raman Scattering of Ultrathin $SnSe_2$ Nanoflakes by Chemical Vapor Deposition

Yongheng Zhang [1], Ying Shi [1], Meimei Wu [1], Kun Zhang [1], Baoyuan Man [1] and Mei Liu [1,2,*]

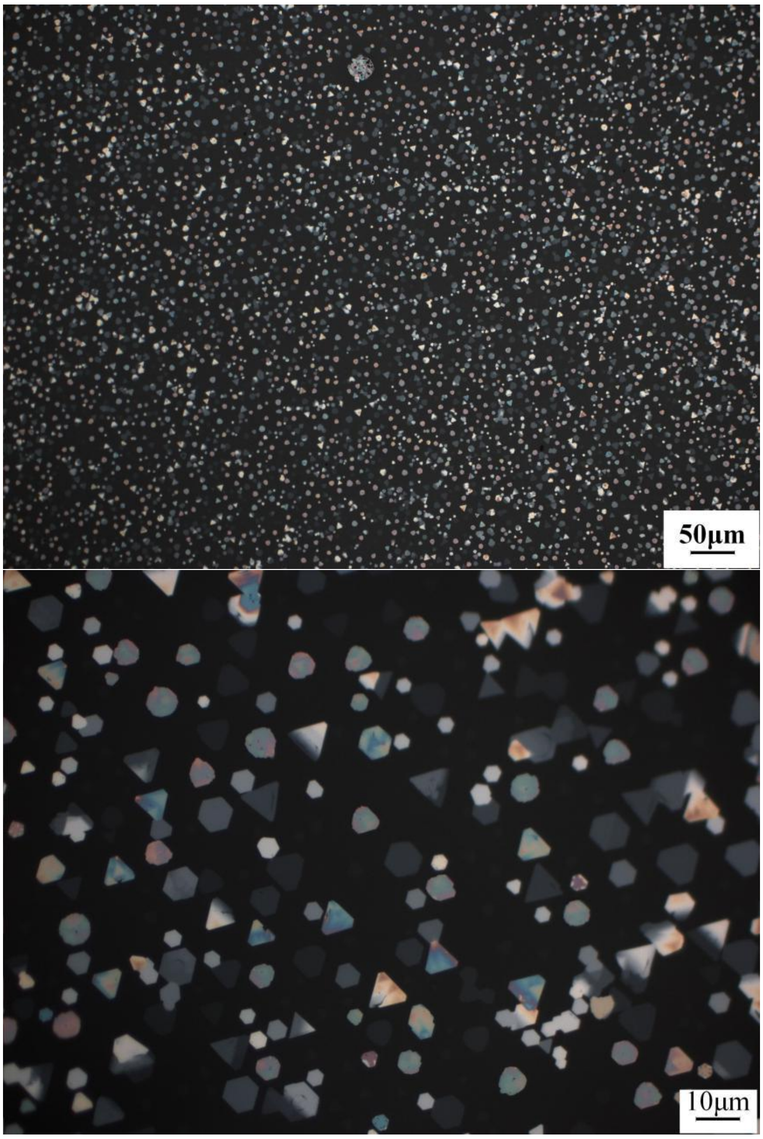

Figure S1. Large-scale optical images of the as-synthesized $SnSe_2$ nanoflakes.

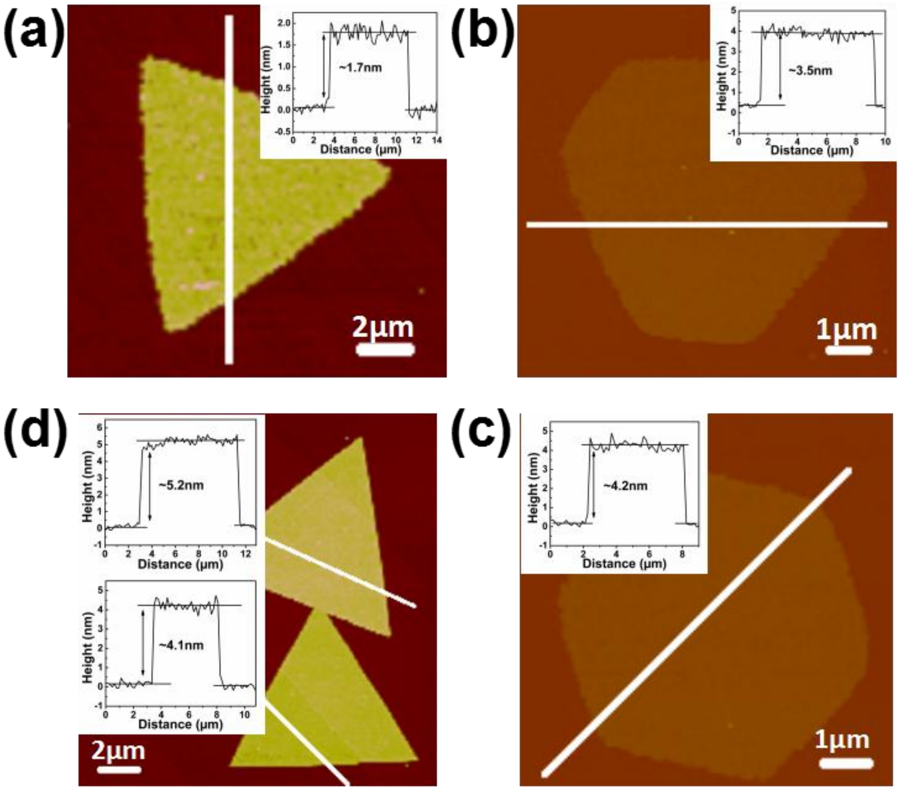

Figure S2. AFM images of $SnSe_2$ nanoflakes with different thickness. The insets show the corresponding height profiles.

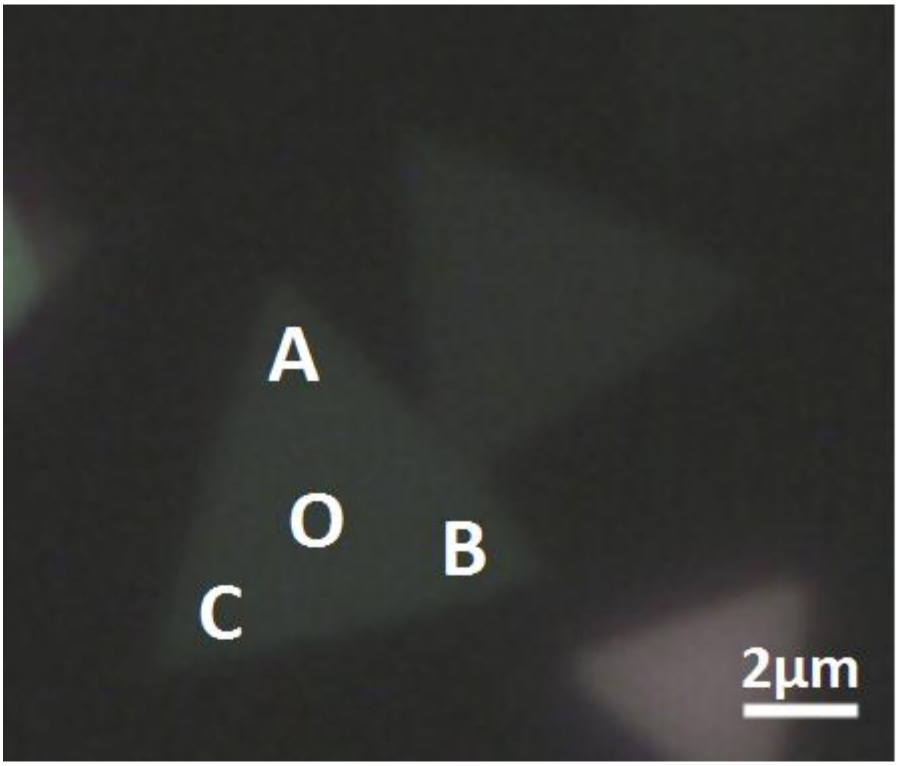

Figure S3. The Raman optical image of triangular nanoflakes, The Raman spectra corresponding to four positions are found in Figure 4c.

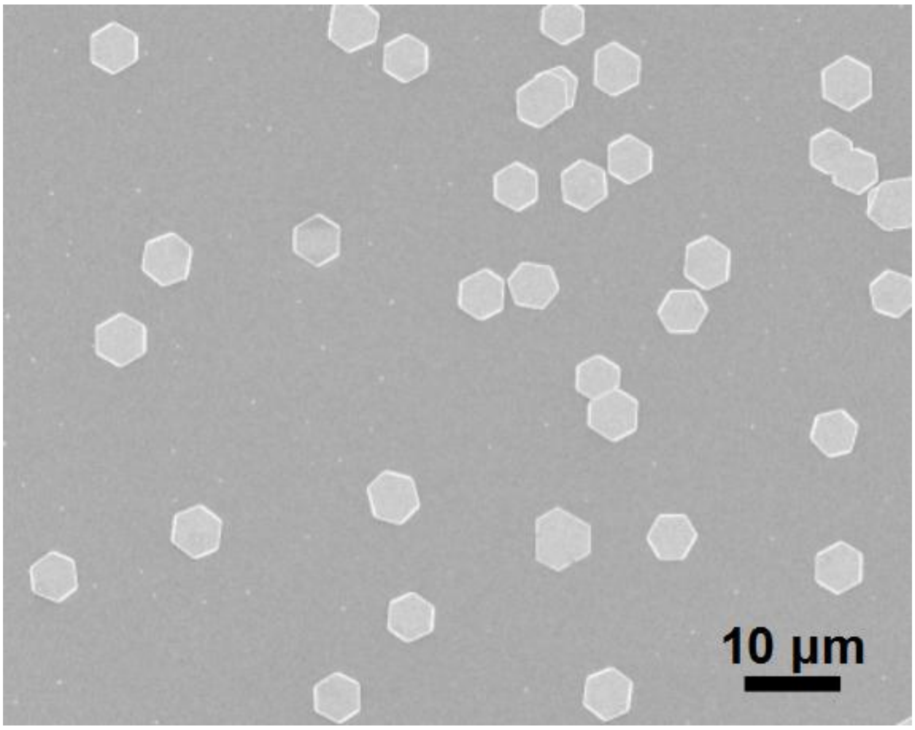

Figure S4. SEM image of $SnSe_2$ nanoflakes in higher pressure.
